# Curcumin Nanoparticles Attenuate Lipotoxic Injury in Cardiomyocytes Through Autophagy and Endoplasmic Reticulum Stress Signaling Pathways

**DOI:** 10.3389/fphar.2021.571482

**Published:** 2021-03-11

**Authors:** Yue Gu, Huan Xia, Xiao Chen, Jing Li

**Affiliations:** ^1^ Department of Reparatory and Critical Care Medicine, The First Affiliated Hospital of Jilin University, Changchun, China; ^2^ Hubei Province Key Laboratory on Cardiovascular, Cerebrovascular, and Metabolic Disorders, Hubei University of Science and Technology, Xianning, China; ^3^ Medical College, Huzhou University, Huzhou, China

**Keywords:** curcumin, nanoparticles, cardiomyocyte, autophagy, endoplasmic reticulum stress

## Abstract

Although curcumin (CUR) has many advantages, its hydrophobicity and instability limit its application. In this study, the anti-lipotoxic injury activity of CUR-loaded nanoparticles (CUR-NPs) and the corresponding mechanism were examined in palmitate (PA)-treated cardiomyocytes. An amphiphilic copolymer was selected as the vehicle material, and CUR-NPs with suitable sizes were prepared under optimized conditions. Cellular uptake was examined by confocal laser scanning microscopy, and cell proliferation inhibition rate was measured using the 3-(4,5-dimethylthiazol-2-yl)-2,5-diphenyltetra bromide (MTT) assay. The terminal deoxynucleotidyl transferase-mediated dUTP nick end labeling (TUNEL) assay was used to detect cell apoptosis. The protein expression was detected by western blot. Exposure to PA reduces the proliferation of cardiomyocytes, but this effect was strongly reversed by CUR-NPs. In addition, our data showed that CUR-NPs strongly inhibited cell apoptosis in PA-treated cardiomyocytes. Furthermore, CUR-NPs remarkably increased the expression of LC3-II, as well as inhibited the expression of p-PERK, *p*-eIF2α, and ATF4 in PA-treated cardiomyocytes. Salubrinal (an eIF2α inhibitor) blocked the protective effect of CUR-NPs against PA-induced cardiomyocyte injury. Our results suggested that CUR-NPs can activated the autophagy pathway and protect myocardial cells from apoptosis, and these effects may be mediated by the eIF2α-related endoplasmic reticulum stress signaling pathway.

## Introduction

The incidence of obesity has been increasing, and obesity has adverse effects on cardiovascular hemodynamics, structure, and function, thus increasing the prevalence of most cardiovascular diseases (Sun et al., 2018). It has been reported that cardiovascular disease is associated with plasma free fatty acid (FFA) levels (Ghosh et al., 2017). FFAs affect lipid and glucose metabolisms, which may exacerbate atherosclerosis and insulin resistance (Ishida et al., 2015). Therefore, FFAs are considered a risk indicator for diabetes, obesity, and cardiovascular diseases (Gu et al., 2018). Many studies have confirmed that heart disease caused by obesity is associated with increased FFA levels (Storniolo et al., 2014; Kamleh et al., 2018).

The pathological damage of FFAs to the heart and their molecular mechanisms are related to many factors. Patients with triglyceridemia generally have excessive intake of FFAs and increased circulating levels of FFAs, which can induce enhanced oxidation and storage of triglycerides or accumulation of toxic lipid species (Xiong, 2018). This may ultimately lead to lipotoxic damage to myocardial cells, including apoptosis and pathological autophagy (Law et al., 2018; Zhang et al., 2018). Recently, many studies have confirmed that autophagy plays a key role in the regulation of cell function (Bartolomé et al., 2017). Autophagy regulation can remove misfolded proteins or damaged organelles to maintain physiological balance *in vivo* (Klionsky and Emr, 2000). However, pathological autophagy is also a direct cause of cell death (Gurusamy and Das, 2009). In recent years, pathological autophagy has also been considered a molecular mechanism of lipotoxic damage (Nissar et al., 2017). It has also been reported that autophagy is related to regulation of the endoplasmic reticulum stress (ERS) signaling pathway (Nissar et al., 2017). In addition, FFAs have been reported to stimulate ERS in H9c2 cardiomyocytes (Zou et al., 2017). Because ERS plays an important role in the pathogenesis of lipotoxic cardiomyopathy, there is increasing interest in the use of an anti-ERS agent as a compensatory treatment for lipotoxic myocardial injury.

Curcumin (CUR) belongs to a class of turmeric rhizome extracts. It has various beneficial pharmacological effects, such as anti-diabetic, anti-inflammatory, anti-tumor, and anti-oxidant effects, and has received widespread attention (Pawar et al., 2015; Chakraborty et al., 2017). It is reported that CUR can treat many chronic diseases, including various cancers, cardiovascular diseases, and autoimmune diseases (Jiang et al., 2017; Gawde et al., 2018). Many studies have confirmed that the protective effect of CUR on myocardial cell glucotoxicity is achieved through inhibition of nicotinamide adenine dinucleotide phosphate (NADPH)-mediated oxidative stress, and this protective effect may be related to the PI3K/Akt signaling pathway (Yu et al., 2016). Moreover, CUR reduces cardiomyocyte remodeling and improves cardiac insufficiency in diabetic rats (Ren and Sowers, 2014). These studies fully revealed the potential protective role of CUR in the pathogenesis of metabolic cardiomyopathy. However, CUR has poor water solubility and is easily degraded (Lim et al., 2018). Therefore, the low bioavailability of CUR limits its application. In recent years, nanotechnology has received increasing attention as an effective technical means to increase the solubility and bioavailability of hydrophobic drugs (Sivasami and Hemalatha, 2018).

In recent years, nanoparticles (NPs) with hydrophilic blocks on their surface have been widely used to deliver hydrophobic drugs *in vivo* because these NPs help to extend the circulation time of hydrophobic drugs in the body, ultimately increasing the biological utilization and efficacy of the drugs (Brahmachari et al., 2011). In our study, monomethoxypoly (ethylene glycol)-b-poly (dl-lactide; PEG-PDLLA) was selected as a carrier to deliver CUR, and it was autonomously loaded into CUR-NPs. We established a palmitate (PA)-induced cardiomyocyte lipotoxicity model, evaluated the protective effect of CUR-NPs on lipotoxicity, and investigated whether the protective mechanism of CUR-NPs is related to the ERS/autophagy signaling pathway. To determine whether CUR-NPs inhibit lipotoxic injury to cardiomyocytes, cytotoxicity was evaluated using the 3-(4,5-dimethylthiazol-2-yl)-2,5- diphenyltetra bromide (MTT) assay. Cellular uptake was measured by confocal laser scanning microscopy (CLSM). Cardiomyocyte apoptosis was examined by the terminal deoxynucleotidyl transferase-mediated dUTP nick end labeling (TUNEL) assay. The changes of ERS/autophagy signaling pathway-related proteins and apoptosis-related proteins were detected by western blotting analysis.

### Experimental Methods

#### Chemical Materials

H9c2 cardiomyocytes were procured from the Cell Bank of the Type Culture Collection of the Chinese Academy of Sciences (China). Dulbecco’s modified Eagle’s medium (DMEM), fetal bovine serum, and type-II collagenase were purchased from Gibco; Thermo Fisher Scientific, Inc. A TUNEL detection kit was purchased from Roche Diagnostics. Rabbit polyclonal antibodies against Bcl-2, Bax (Bcl-2-associated X protein), protein kinase R-like ER kinase (PERK), phosphorylated PERK (p-PERK), eukaryotic translation initiation factor 2α (eIF2α), phosphorylated eIF2α (*p*-eIF2α), activating transcription factor 4 (ATF4), autophagy-related protein LC3, and β-actin were purchased from Cell Signaling Technology, Inc. Hybond C membranes and ECL western blot detection kit were purchased from Pierce; Thermo Fisher Scientific, Inc. An MTT assay kit, PA, and salubrinal were purchased from Sigma-Aldrich; Merck KGaA. PEG-PDLLA (PEG, 5 kDa; PDLLA, 10 kDa) was purchased from Advanced Polymer Materials, Inc (Canada). PA and all other reagents were of analytical grade and used as received.

#### Preparation of PA

PA was dissolved in 0.1 M NaOH solution in a 70°C water bath. After shaking and mixing for 10 min, the PA storage solution of 100 mM was prepared by filtration. In 55°C water bath, 50 g/L bovine serum albumin (BSA) solution was prepared with deionized water and filtered. The PA/BSA composite solution was prepared by mixing the PA solution and BSA solution in the volume ratio of 1:19. The PA/BSA composite solution was shaken in the water bath for 10 s, and then the water bath was continued for 10 min after being taken out, it was cooled to room temperature and filtered. Then, the compound solution was diluted with DMEM medium.

#### Synthesis and Characterization of CUR-NPs

PEG-PDLLA copolymers were synthesized according to previously reported methods (Brahmachari et al., 2011). CUR-NPs were prepared as follows. Briefly, under vigorous stirring at 25°C, CUR (1 mg) and PEG-PDLLA (9 mg) were dissolved in tetrahydrofuran and dropped into 10 ml distilled water. The resulting mixture was dialyzed to obtain CUR-NPs. To calculate the drug loading (DL) and drug loading efficiency (LE) of CUR-NPs, CUR-NPs were dispersed in methanol and sonicated to extract CUR. CUR content in CUR-NPs was measured by high-performance liquid chromatography (Eclipse XDB-C18 column; 150 × 4.6 mm; 5 μm; Agilent Technologies, Inc.) under the following operating conditions: the mobile phase was methanol containing 3 mM potassium monohydrogen phosphate and acetic acid (methanol/potassium hydrogen phosphate/acetic acid/water = 230/20/2/748, v/v); flow rate, 1.0 ml/min; injection volume, 20 μL; column temperature, 25°C; detection wavelength, 227 nm. DL and LE were calculated using following formulas:

$$\text{DL}\cdot \left(\%\right)=\left(M_{0}/M\right)\times 100\%,$$ (1)

$$\text{LE}\cdot \left(\%\right)=\left(M_{0}/M_{1}\right)\times 100\%,$$ (2)

In the above formula, the mass of CUR in NPs is represented by *M*_*0*_, the mass of CUR in feed is represented by *M*_*1*_, and the mass of NPs is represented by *M*.

The morphological characteristics of the NPs were observed by transmission electron microscopy (TEM; Tecnai G2-20). The zeta potential of NPs was measured using a Nano-ZS instrument (Malvern, United Kingdom). The size of the NPs was measured by dynamic light scattering (Wyatt Technology, United States).

#### Release of CUR From NPs

2 ml phosphate-buffered saline (PBS; pH 7.4, containing 1.0 wt% Tween-80) was taken to dissolve CUR-NPs and put into dialysis bag (MW cut-off value: 3.5 kDa). The dialysis bag was put into 8 ml release medium and cultured in 37°C water bath under shaking. At a certain interval, 1 ml of liquid from the dialysis bag was removed for UV-vis analysis and replaced with 1 ml of fresh buffer solution. The absorbance at 427 nm was detected, and the CUR released by NPs in each incubation time period was calculated through the calibration curve, and the cumulative CUR release was finally obtained.

#### Cell Culture and Drug Treatment

Double antibody and 10% fetal bovine serum were added into DMEM medium to culture H9c2 cardiomyocytes. When the cell adhesion density was between 70% and 80%, trypsin containing 0.25% EDTA was used to digest and subculture H9c2 cardiomyocytes. The cells were then treated with 0.2 mM PA for 24 or 48 h to establish a cytotoxicity model. CUR-NPs were added at 1 h before the addition of a PA-containing medium. The cells were pretreated with 30 μM sarubulin for 1 h, then CUR-NPs was added. The control group was given the same volume of PBS.

#### Cell Uptake

Cellular uptake was measured by CLSM. Sterile coverslips were placed in a six-well plate, and the cells were cultured at 37°C for 24 h until 2×10^4^ cells adhered to each well. After the addition of CUR-NPs (CUR equivalent, 100 μM) to each well, the cells were incubated for 1 h. Remove the supernatant and wash the precipitate three times with PBS. Next, 800 μL of 4% formaldehyde was added to each well to fix the cells, which were subsequently incubated at 25°C for 20 min and washed again twice with PBS. To examine cell localization, the nuclei were stained with 4′,6-diamidino-2-phenylindole. Finally, the cells on the slides were observed by CLSM.

#### Cytotoxicity Test

The 96-well plate was used to inoculate cells with a density of 1×10^5^ cells/well. Each well was added to a culture solution containing the corresponding drug. The cells were incubated at 37°C for 48 h. At the end of experiment, remove the supernatant and add 20 μL of MTT solution (5 mg/ml in PBS) to the cells in each well. The cells were incubated at 37 °C and 5% CO_2_ for 4 h and then removed from the supernatant. Next, 150 μL of dimethyl sulfoxide (DMSO) was added to cells, and the absorbance at 570 nm was measured using a microplate reader (Bio-Rad, United States).

#### TUNEL Analysis

Morphological detection of apoptosis was performed with TUNEL detection kit (Millipore, Billerica, MA, United States). The number of TUNEL positive cells was detected by flow cytometry and fluorescence microscope respectively to evaluate the apoptosis of each group (Olympus IX71; Olympus, Japan). The ratios of TUNEL-positive cells in the test and control groups were analyzed. Refer to the flow cytometry method provided in reference to detect apoptosis (Sun et al., 2015).

#### Western Blot Analysis

The cells cultured in the six-well plates were collected, lyzed on ice for 30 min, and centrifuged (12,000×*g* and 4°C) for 15 min. The supernatant was removed and the precipitate was obtained for protein quantification and sample preparation. Briefly, 20 μL of samples was loaded to 5–15% concentrated gel for protein electrophoresis separation. After electrophoresis, the protein was transferred from the gel to the PVDF membrane, which were then blocked using 5% skim milk powder for 1 h at 25°C. The membranes were then incubated with a primary antibody at 37°C for 24 h, washed with PBS, further incubated with a secondary antibody for 1 h, and then visualized by electrogene-rated chemiluminescence. The expression of the following proteins was detected using a gel imaging system: Bcl-2, Bax, LC3, p-PERK, PERK, *p*-eIF2α, eIF2α, ATF4 (Cell Signaling Technology), and β-actin (Santa Cruz Biotechnology).

#### Statistical Analysis

The data were expressed as mean ± standard deviation. The GraphPad Prism v5 software (GraphPad Software, Inc.) was used for statistical analysis of the data. *t* test and one-way ANOVA analysis were used to compare the differences between groups.

## Results

### Characterization Data of CUR-NPs

**FIGURE 1:**
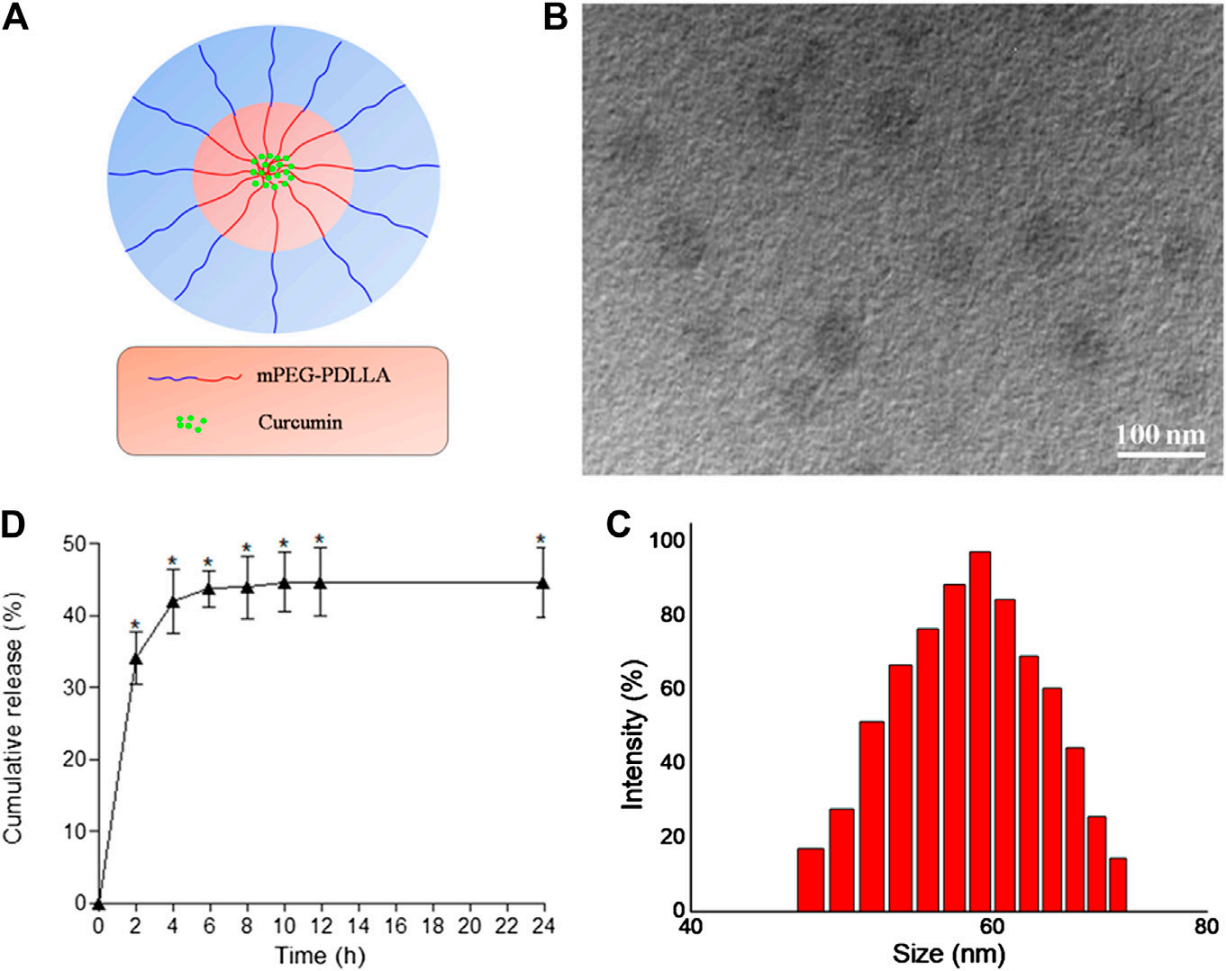
Characterization of CUR-NPs **(A)** The structural scheme of CUR-NPs **(B)** TEM image of CUR-NPs **(C)** Size distribution of CUR-NPs determined by DLS **(D)** Cumulative release profiles of CUR from CUR-NPs in PBS (pH 7.4) for 24 h.

We successfully prepared desirable CUR-NPs through optimal assembly conditions. Figure 1A shows our structural scheme. The transmission electron microscopy image of the obtained CUR-NPs is shown in Figure 1B. As shown in Figure 1B, CUR-NPs had the following characteristics: round-shaped NPs smaller than 100 nm with uniform size and good dispersion. Figure 1C shows that the average particle size of CUR-NPs is 59.02 ± 13.06 nm (polydispersity index, 0.28), and its distribution shows the characteristics of Gaussian distribution. The zeta potential of CUR-NPs was also assessed; the results showed that it was nearly neutral and approximately 0.48 ± 0.017 mV. In addition, analysis of CUR-NPs revealed a DL of 8.59%, LE of 82.5% ± 0.63%, and critical aggregation concentration of 0.05 ± 0.0027 mg/ml. Based on the above data, CUR-NPs had a small size and a clinically acceptable critical aggregation concentration; thus, they had practical application value.

We tested CUR release in a 37°C buffer solution containing CUR-NPs. As shown in Figure 1D, the release curve of CUR was very steep in the first 4 h, with the release amount quickly reaching approximately 41.9%. After 4 h, CUR release became slow and smooth, which may be caused by the strong hydrophobicity of CUR.

### Intracellular Uptake and Cytotoxicity of CUR-NPs

**FIGURE 2:**
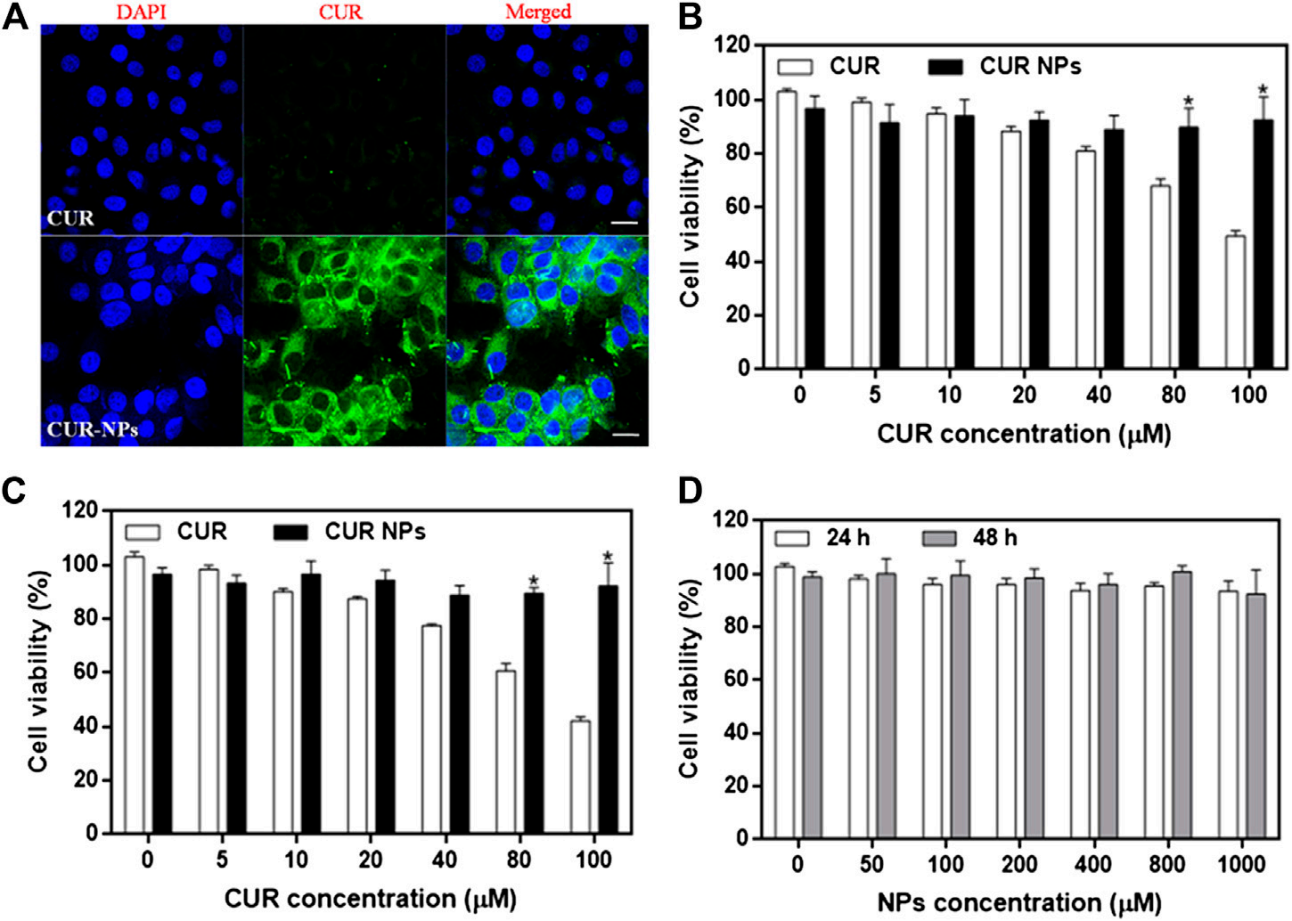
Cellular uptake and cytotoxicity of CUR-NPs on cardiomyocytes **(A)** CLSM images of CUR and CUR-NPs taken by cardiomyocytes. Scale bar 50 μm **(B)** The effect of CUR and CUR-NPs on the viability of cardiomyocytes after 24 h stimulation. *p < 0.05 *vs* CUR group **(C)** The effect of CUR and CUR-NPs on the viability of cardiomyocytes after 48 h stimulation. *p < 0.05 *vs* CUR group **(D)** Effect of blank nanocarrier on viability of cardiomyocytes.

To examine the cellular uptake of CUR-NPs, we measured the uptake of CUR or CUR-NPs by H9c2 cardiomyocytes by CLSM imaging. Our results showed that after 1 h of incubation, the fluorescence intensity of the CUR-NPs group was significantly stronger than that of the CUR group (Figure 2A). This result confirmed that CUR-NPs, compared with small-molecule CUR, significantly increased the cellular uptake of CUR. To determine the effect of CUR and CUR-NPs on the proliferation of H9c2 cardiomyocytes and the range of their toxic concentrations, the viability of H9c2 cardiomyocytes treated with different concentrations of CUR or CUR-NPs for 24 and 48 h was examined. The apoptosis degree of cardiomyocytes was positively correlated with the incubation concentration and incubation time of CUR. When CUR concentration was increased to more than 40 μM, cell viability decreased to below 80% at 24 h (Figure 2B), and the decrease in cell viability was more pronounced at 48 h (Figure 2C). However, CUR-NPs did not cause significant damage to cardiomyocytes. Even when the CUR equivalent was increased to a high concentration of 100 μM and the incubation time was extended to 48 h, there was no significant damage to the cell viability, which reached more than 90% (Figures 2B,C). As shown in Figure 2D, after treatment with different concentrations of blank carriers (NPs), the cell viability of H9c2 cardiomyocytes did not decrease in a concentration-dependent manner at 24 or 48 h. The above results suggested that compared with free CUR, nanometerized CUR prevented the toxicity of high CUR concentration to myocardial cells, thereby improving the efficacy and reducing the toxic effect of CUR.

### CUR-NPs Inhibited PA-Induced H9c2 Cardiomyocyte Injury

**FIGURE 3:**
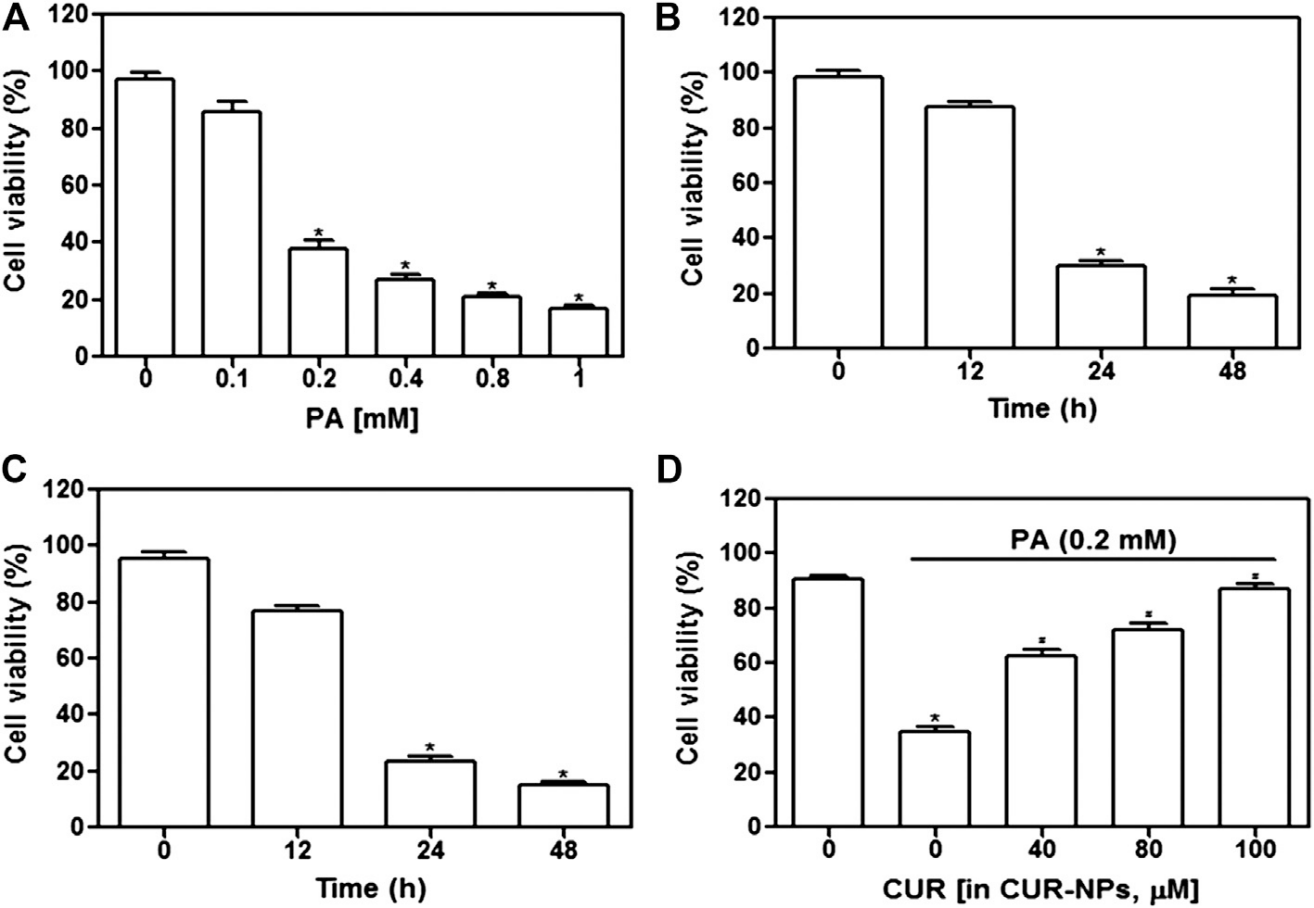
Effect of CUR-NPs on PA-induced cardiomyocyte survival depression **(A)** Effects of different concentrations of PA on viability of cardiomyocytes. *p < 0.05 *vs* 0 mM group **(B)** Changes of viability of cardiomyocytes stimulated with 0.2 mM PA for different time. *p < 0.05 *vs* 0 h group **(C)** Changes of viability of cardiomyocytes stimulated with 0.4 mM PA for different time. *p < 0.05 *vs* 0 h group **(D)** Effect of CUR-NPs on viability of cardiomyocytes stimulated by PA (incubation concentration was 0.2 mM, incubation time was 24 h). *p < 0.05 *vs* control group; ^#^ *p* < 0.05 *vs* PA group.

First, we examined whether high-fat inhibited cardiomyocyte survival. The concentration range of PA was set to 0–1 mM, and the stimulation time was 24 or 48 h. As shown in Figure 3A, cell survival was significantly inhibited when PA concentration was increased to 1 mM (*p* < 0.05) and when the incubation time exceeded 24 h with 0.2 mM PA (*p* < 0.05, Figure 3B) or 0.4 mM PA (*p* < 0.05, Figure 3C). As shown in Figure 3D, the addition of CUR-NPs (CUR equivalent, 100 μM) significantly increased cell viability (*p* < 0.05). These results suggested that CUR-NPs protected cardiomyocytes against lipotoxicity. Moreover, in the following experiments, 0.2 mM PA was used to induce high fat conditions, and CUR-NPs (CUR equivalent, 100 μM) were applied.

### CUR-NPs Abrogated PA-Induced Apoptosis in H9c2 Cardiomyocytes

**FIGURE 4:**
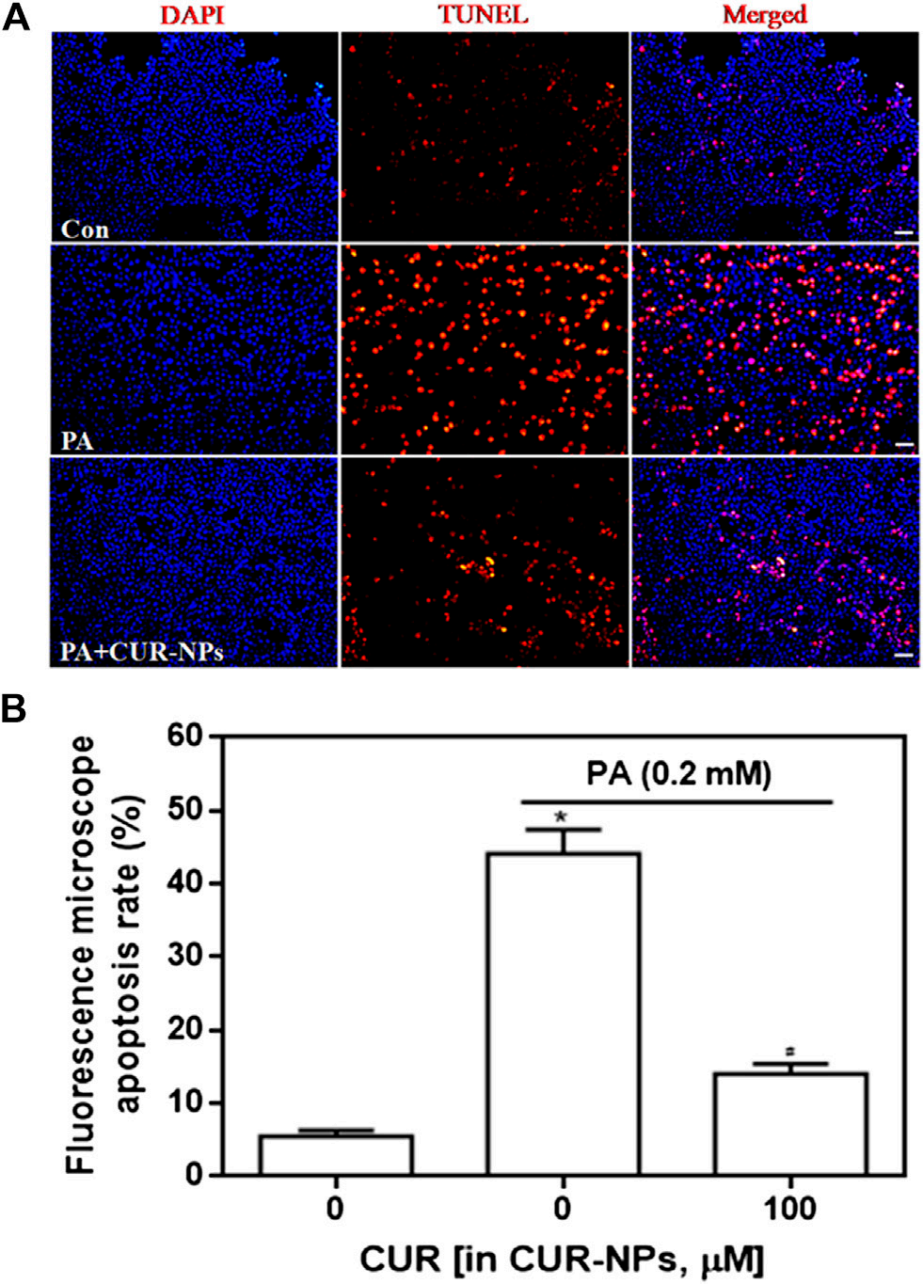
Effect of CUR-NPs on PA-induced cardiomyocyte apoptosis **(A)** TUNEL fluorescence staining image data of cardiomyocyte apoptosis (bar is 20 μm) **(B)** Quantitative analysis for TUNEL staining by fluorescence microscopy. *p < 0.05 *vs* control group; ^#^ *p* < 0.05 *vs* PA group.

**FIGURE 5:**
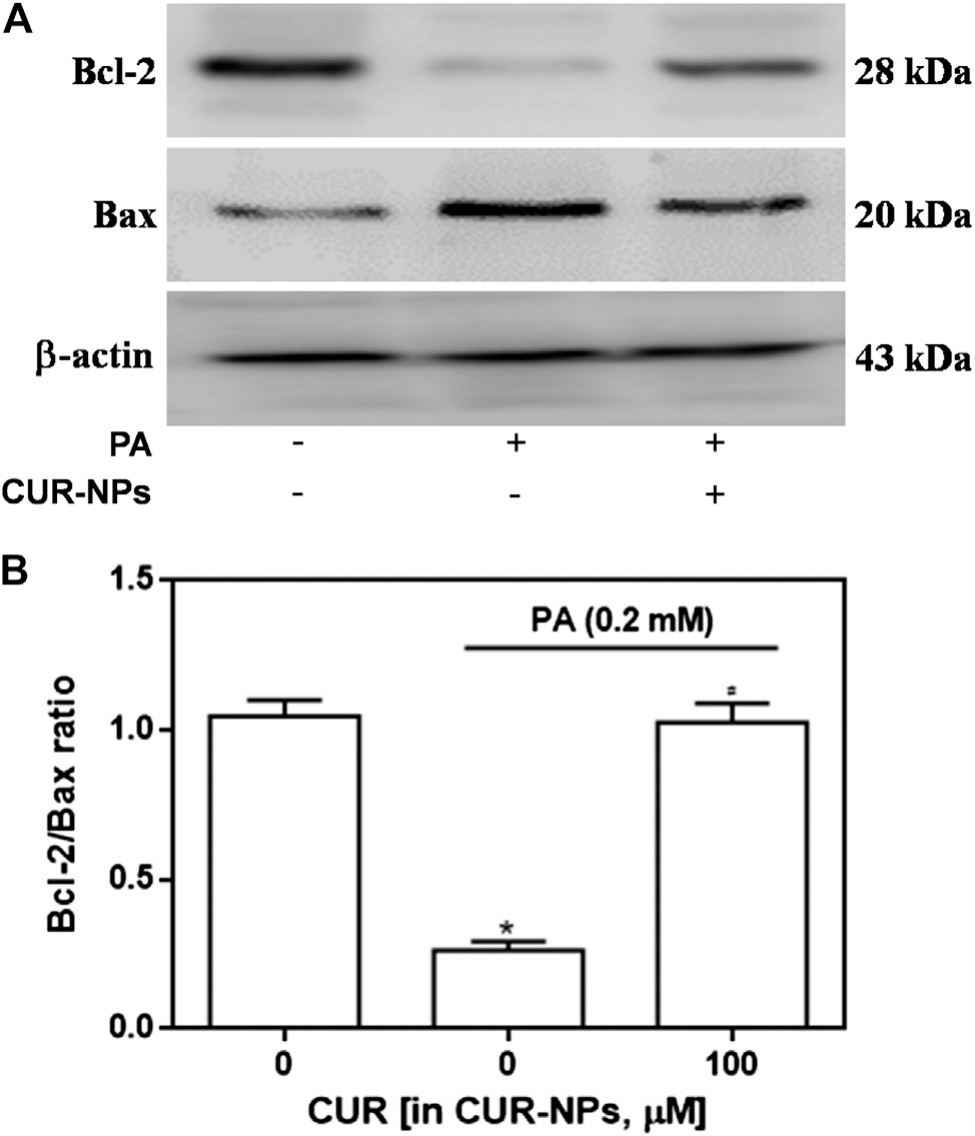
Effects of CUR-NPs on the expression of apoptosis pathway-related proteins in cardiomyocytes exposed to PA **(A)** Representative images of Bax and Bcl-2 expression by western blot **(B)** Quantitative analysis of the Bcl-2/Bax ratio. *p < 0.05 *vs* control group; ^#^ *p* < 0.05 *vs* PA group.

Apoptosis was detected by the TUNEL assay, and red fluorescence in Figure 4A indicates apoptotic cells. Our results suggested that PA significantly increased apoptosis, compared with that in the control group, whereas CUR-NPs significantly inhibited PA-induced apoptosis, as shown by the apparent reduction or disappearance of intracellular red fluorescence (*p* < 0.05, Figure 4B). Apoptosis is accomplished through the apoptotic pathway, which involves the expression of many proteins (Law et al., 2018). Therefore, we examined the expression of apoptotic pathway-related proteins by immunoblotting to prove the correlation between the protective effect of CUR-NPs on cardiomyocytes is related to anti-apoptosis. The results showed that PA increased the level of Bax and decreased the level of Bcl-2. Bax was a pro-apoptotic protein and Bcl-2 was an anti-apoptotic protein. The results suggested that PA could significantly promote the apoptosis of cardiomyocytes (Figure 5A). This effect was successfully reversed by CUR-NPs (CUR equivalent, 100 μM), as it decreased Bax expression and increased Bcl-2 expression (Figure 5A). Statistical analysis showed that the significant decrease of Bcl-2/Bax ratio in PA group was effectively abolished by CUR-NPs, which was consistent with the results of band analysis (*p* < 0.05, Figure 5B).

### Effect of CUR-NPs on Autophagy in H9c2 Cardiomyocytes Exposed to PA

**FIGURE 6:**
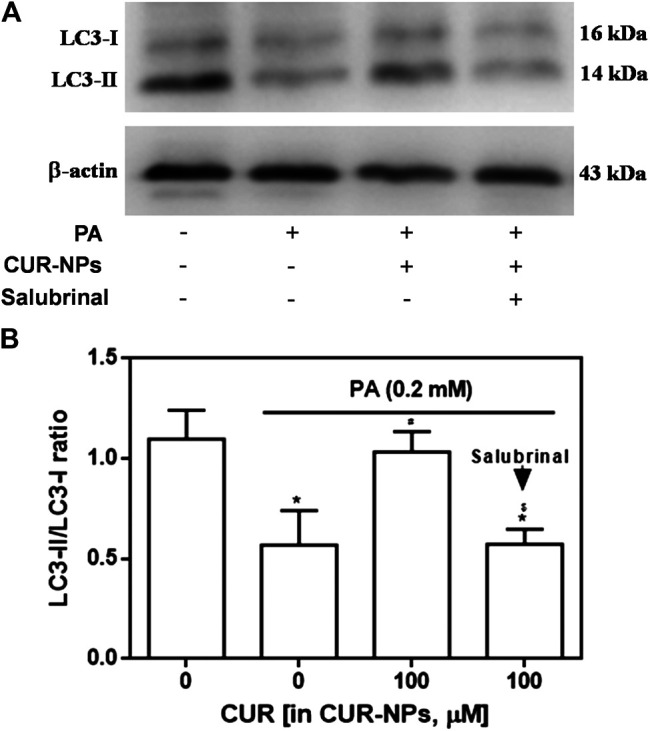
Effects of CUR-NPs on the expression of autophagy pathway-related proteins in cardiomyocytes exposed to PA **(A)** Representative images of LC3-I and LC3-II expression by western blot **(B)** Quantitative analysis of the LC3-II/LC3-I ratio. Salubrinal: 30 μM *p < 0.05 *vs* control group; ^#^ *p* < 0.05 *vs* PA group; ^$^ *p* < 0.05 *vs* PA + CUR group.

As shown in Figure 6A, compared with the control group, the expression of LC3-II in PA group was significantly decreased, but the expression of LC3-I had no significant change, which was abolished by CUR-NPs to the level of control group. As shown in Figure 6B suggested that H9c2 cardiomyocytes treated with PA showed significantly decreased ratio of LC3-II/LC3-I. However, the decrease in the ratio of LC3-II/LC3-I in PA-treated H9c2 cardiomyocytes was abolished by CUR-NPs (*p* < 0.05, Figure 6B). These data indicated that the anti-lipotoxicity effect of CUR-NPs was mainly achieved through activation of the autophagy signaling pathway. Moreover, as shown in Figure 6B, the ratio of LC3-II/LC3-I increased by CUR-NPs was also abolished when ERS was inhibited by salubrinal (an eIF2α inhibitor). These results suggested that eIF2α-mediated inhibition of the ERS pathway was critical for CUR-NPs to activated autophagy and prevent apoptosis in cardiomyocytes in response to lipotoxicity injury.

### Effect of CUR-NPs on ERS Signaling in H9c2 Cardiomyocytes Exposed to PA

**FIGURE 7:**
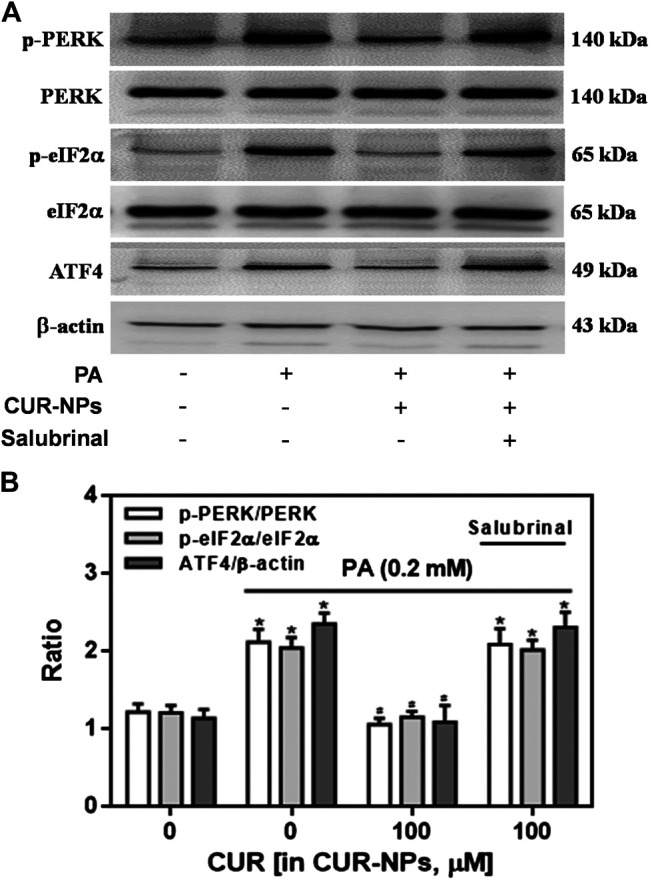
Effects of CUR-NPs on the expression of PERK/eIF2α/ATF4 pathway-related proteins in cardiomyocytes exposed to PA **(A)** Representative images of p-PERK, PERK, *p*-eIF2α, eIF2α, ATF4 and β-actin expression by western blot **(B)** Quantitative analysis of the p-PERK/PERK, *p*-eIF2α/eIF2α and ATF4/β-actin. Salubrinal: 30 μM *p < 0.0*5 vs* control group; ^#^ *p* < 0.05 *vs* PA group; ^$^ *p* < 0.05 *vs* PA + CUR group.

**FIGURE 8:**
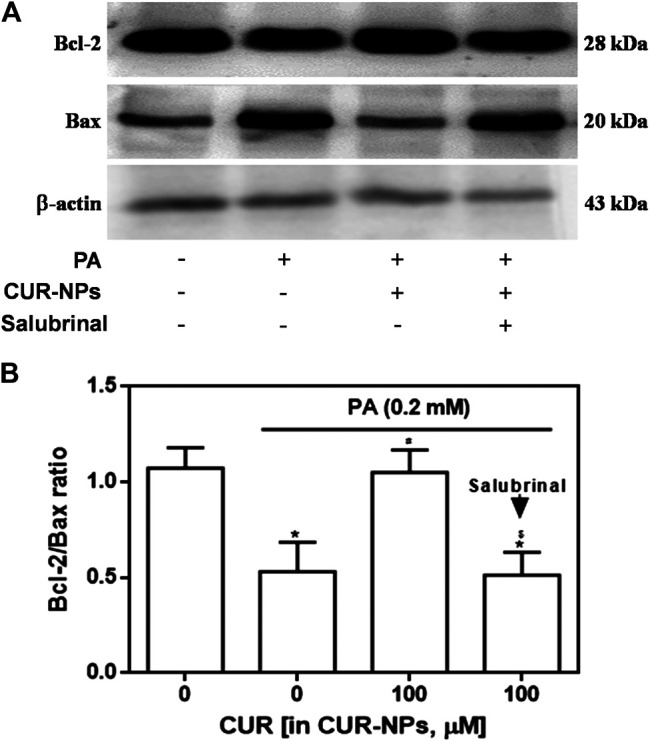
Effect of salubrinal pretreatment on the anti-apoptotic effect of CUR-NPs in cardiomyocytes with lipotoxic injury **(A)** Representative images of Bax and Bcl-2 expression by western blot **(B)** Quantitative analysis of the Bcl-2/Bax ratio. Salubrinal: 30 μM *p < 0.05 *vs* control group; ^#^ *p* < 0.05 *vs* PA group; ^$^ *p* < 0.05 *vs* PA + CUR group.

It has been confirmed that ERS pathway is involved in the lipid toxicity of hyperlipidemia to cardiomyocytes (Zou et al., 2017). In the current experiment, we tested the expression of ERS-related proteins to prove the correlation between the protective effect of CUR-NPs on PA-induced apoptosis and the ERS pathway in cardiomyocytes. As shown in Figure 7A, compared with the control group, the expressions of total PERK and total eIF2α in PA group were not significantly changed, but the expressions of p-PERK, *p*-eIF2α and ATF4 in PA group were significantly increased (*p* < 0.05), which was completely inhibited by CUR-NPs (*p* < 0.05). The results in Figure 7B suggest that CUR-NPs can successfully counteract the abnormal changes of ERS pathway proteins induced by PA, which is consistent with the results in Figure 7A. Pretreatment with Salubrinal (30 μM), an inhibitor of ERS pathway mediated by eIF2α, could eliminate the ERS regulation effect of CUR-NPs (*p* < 0.05). Moreover, in PA-treated H9c2 cardiomyocytes, pretreatment with salubrinal blocked the regulatory effect of CUR-NPs on Bax and Bcl-2 expression (Figure 8A). As shown in Figure 8B, compared with the control group, the ratio of Bcl-2/Bax in PA group was significantly decreased, indicating that PA stimulation led to significant apoptosis of cardiomyocytes (*p* < 0.05), which could be effectively abolished by CUR-NPs (*p* < 0.05). Pretreatment with salubrinal could significantly counteract the regulation of Bcl-2/Bax ratio by CUR-NPs (*p* < 0.05). Thus, these results suggested that the protective effect of CUR-NPs on cardiomyocytes was closely related to the eIF2α-mediated ERS pathway.

## Discussion

Lipid toxicity is an important cause of metabolic cardiomyopathy in rodents and humans (Li et al., 2018). Hypertriglyceridemia causes abnormalities in the intake and circulating levels of FFA as well as abnormal degrees of oxidation (Li et al., 2018), ultimately inducing pathological changes, such as myocardial cell apoptosis (Yang et al., 2014). Many studies have suggested that cardiomyocyte apoptosis is closely related to impaired myocardial function. Therefore, regulation of cardiomyocyte apoptosis has been considered an effective approach to improve cardiac function (Bi et al., 2018). Inhibition of cardiomyocyte apoptosis may also be an effective strategy to improve myocardial cell lipotoxicity (Zou et al., 2017). We found that PA significantly decreased cardiomyocyte proliferation and increased cell apoptosis.

It is reported that CUR exerts anti-oxidant effect by reducing the level of oxidative stress, and ultimately inhibits cardiomyocyte apoptosis (Yu et al., 2016). In the present study, CUR was nanometerized to obtain highly water-soluble NPs, which were then used to effectively inhibit lipotoxicity-induced myocardial cell apoptosis, thereby playing a cardioprotective role. According to the TUNEL assay, CUR-NPs reduced apoptosis in PA-treated cardiomyocytes. Moreover, western blotting analysis results suggested that CUR-NPs effectively reversed PA-induced increase in Bax expression and decrease in Bcl-2 expression. Bax is a key regulatory protein in the mitochondrial-mediated apoptosis pathway (Mikhailov et al., 2001), whereas Bcl-2 is an anti-apoptotic protein that organizes Bax oligomerization (Mikhailov et al., 2001). The above results indicated that the protective effect of CUR-NPs on cardiomyocytes was related to the cardiomyocyte apoptosis pathway.

In physiological state, autophagy plays a key role in controlling protein quality and recovering damaged cytoplasmic components (Nanni et al., 2018; Bartolomé et al., 2017). Many studies have confirmed that autophagy is necessary for the maintenance of normal cardiac function (Nanni et al., 2018; Bartolomé et al., 2017). Moreover, other studies indicated that autophagy acts as a protective mechanism of cells under pathological conditions, such as hyperlipidemia (Nissar et al., 2017). Increasing evidence suggests that autophagy plays a role as a critical factor linking hyperlipidemia with its associated complications, such as cardiovascular diseases (Nanni et al., 2018). LC3 (LC3-I) located in the cytoplasm is recruited into autophagosome to form LC3 (LC3-II), which is the key process of autophagy (Notaro et al., 2014). It has also been reported that LC3-II/LC3-I ratio can be an important indicator of autophagy (Lassak et al., 2018). In recent years, it has been reported that lipotoxic injury can inhibit the autophagy level of cardiomyocytes (Ceylan-Isik et al., 2013). In this study, H9c2 cardiomyocytes treated with PA showed significantly decreased ratio of LC3-II/LC3-I at 24 h. However, the decreased ratio of LC3-II/LC3-I in PA-treated H9c2 cardiomyocytes was abolished by CUR-NPs. These data indicated that the cardioprotective effect of CUR-NPs was mainly achieved through activation of the autophagy signaling pathway.

Recent reports suggest that ERS pathway plays a key role in the pathogenesis of hyperlipidemia and its complications (Zou et al., 2017). ERS is an important pathway of apoptosis. The endoplasmic reticulum can accurately sense cell stress and then induce stress-related reactions, including those caused by metabolic and protein folding disorders. ERS can activate the unfolded protein reaction pathway, which is composed of a baroreceptor, such as glucose-regulated protein 78 and PERK protein kinase, and downstream signal transduction molecules (Yang et al., 2015). In the resting state, glucose-regulated protein 78 is combined with the baroreceptor. Under ERS, glucose-regulated protein 78 is expressed in large quantities to maintain endoplasmic reticulum homeostasis, and separated from the baroreceptor. The corresponding unfolded protein reaction signaling pathway is activated to induce the expression of CCAAT/enhancer binding protein and promote cell apoptosis. PERK is mainly composed of two domains, including the domain binding glucose-regulated protein 78 protein and the threonine/serine kinase domain that promotes eIF2α phosphorylation. eIF2α is a downstream signal transduction molecule of PERK protein kinase. Phosphorylated eIF2α activates the downstream ATF4 protein and eventually induces the expression of CCAAT/enhancer binding protein. Salubrinal is an inhibitor of ERS pathway mediated by eIF2α, that is, it can inhibit the activation of ERS pathway by inhibiting PERK/eIF2α/ATF4 pathway (Cnop et al., 2007). Our data confirm that the activation of PERK/eIF2α/ATF4 pathway induced by PA can be effectively inhibited by CUR-NPs in cardiomyocytes, and this effect can be effectively abolished by salubrinal. On the other hand, salubrinal can eliminate the anti-apoptotic effect of CUR-NPs by inhibiting eIF2α−mediated ERS pathway. It has also been reported that the regulation of autophagy is closely related to the activation of ERS pathway (Hoyer-Hansen and Jaattela, 2007). Our results indicated that salubrinal abolished the autophagy-activation effect of CUR-NPs by inhibiting the eIF2α-mediated ERS pathway. These data indicated that the cardioprotective effect of CUR-NPs was mainly achieved through regulation of the eIF2α-mediated autophagy signaling pathway.

## Conclusion

In summary, CUR-NPs significantly inhibited the apoptosis of cardiomyocytes induced by high fat, which may be related to autophagy pathway activation and PERK/eIF2α/ATF4 pathway inhibition *in vitro*. It can be inferred that CUR-NPs may become a new drug dosage form for the treatment of myocardial lipotoxic injury. It is worth considering that blank vector and free curcumin as two variables may weaken the conclusion, which is a limitation of this study, and supplementary experiments need to be done in the future.

## Data Availability

The raw data supporting the conclusion of this article will be made available by the authors, without undue reservation.
